# Colonic Diffuse Large B-Cell Lymphoma Presenting as Fecal Peritonitis: A Case Report

**DOI:** 10.7759/cureus.111609

**Published:** 2026-06-27

**Authors:** Mouad Ouryemchi, Soufiane Taibi, Abdelali Guellil, Rachid Jabi, Mohammed Bouziane

**Affiliations:** 1 Department of General Surgery, Mohammed VI University Hospital, Oujda, MAR; 2 Laboratory of Anatomy, Microsurgery and Surgery Experimental and Medical Simulation (LAMCESM), Faculty of Medicine and Pharmacy, Mohammed First University, Oujda, MAR

**Keywords:** case report, colectomy, colonic b-cell lymphoma, immunochemotherapy, peritonitis

## Abstract

Primary colonic lymphoma is a rare neoplasm. Perforation with fecal peritonitis as the initial manifestation is an exceptional and life-threatening complication requiring emergency surgical management. We report a case of diffuse large B-cell lymphoma of the colon presenting with fecal peritonitis, highlighting the diagnostic challenges and multidisciplinary approach required. A 70-year-old female patient with a history of hypertension presented to the emergency department with an acute abdomen, septic shock, and generalized peritonitis. Abdominal computed tomography (CT) revealed fecal peritonitis secondary to perforation of a cecal tumor. Emergency right oncologic colectomy with double stoma was performed because of generalized purulent peritoneal contamination. Histopathological and immunohistochemical examination confirmed diffuse large B-cell lymphoma, germinal center type, with a high proliferation index (antigen Ki-67, 90%), cluster of differentiation 79a (CD79a)-positive, and cluster of differentiation 3 (CD3)-negative. The postoperative course was uneventful after a two-day intensive care unit (ICU) stay. The patient received four cycles of immunochemotherapy based on rituximab, cyclophosphamide, doxorubicin, vincristine, and prednisone (R-CHOP). At six months, PET-CT evaluation demonstrated a partial response, and treatment is ongoing to complete six cycles. This case illustrates that colonic lymphoma should be considered in the differential diagnosis of acute abdomen with tumor perforation. Emergency colectomy serves both diagnostic and therapeutic purposes by controlling the septic source and providing tissue for definitive histological diagnosis. Multidisciplinary management combining emergency surgery and adjuvant immunochemotherapy is essential for optimizing outcomes in this rare presentation.

## Introduction

Primary gastrointestinal lymphomas account for 30-45% of extranodal non-Hodgkin lymphomas, with the stomach being the most common site (50-60%), followed by the small intestine (20-30%) and the colon (10-20%) [1]. Primary colonic lymphomas are exceptionally rare, representing less than 1% of all colonic malignancies [1,2]. Although primary colonic lymphoma remains rare, its incidence is increasing in elderly and immunocompromised populations, and perforation as the initial presenting feature is exceptional. The clinical presentation is typically nonspecific, including abdominal pain, changes in bowel habits, weight loss, or a palpable mass, often leading to delayed diagnosis [3]. Acute complications such as obstruction, intussusception, hemorrhage, or perforation are unusual at initial presentation [4]. Tumor perforation with fecal peritonitis as the presenting manifestation of colonic lymphoma is an exceptional event, with only scattered case reports in the literature [5]. Perforation may result from transmural tumor infiltration leading to wall fragility, tumor necrosis, or obstruction with proximal distension [6]. This life-threatening complication carries a mortality rate exceeding 50% and requires urgent surgical intervention [7]. Emergency colectomy serves a dual purpose: source control of sepsis and procurement of tissue for definitive histopathological diagnosis [8]. We report a case of diffuse large B-cell lymphoma of the colon presenting with fecal peritonitis, emphasizing the diagnostic challenges, surgical management, and importance of a multidisciplinary approach.

## Case presentation

A 70-year-old woman presented to the emergency department with a two-day history of acute abdomen and generalized abdominal pain, with no associated vomiting or cessation of transit. She had a history of arterial hypertension under treatment and no previous surgery. On admission, she presented with systolic arterial hypotension of 80 mmHg, tachycardia of 120 bpm, dehydration, and a fever of 39°C. Physical examination revealed abdominal guarding. The patient had a WHO performance status of 1, a body mass index (BMI) of 24.5 kg/m², and good nutritional status with no recent weight loss. Laboratory tests showed a severe inflammatory reaction with hyperleukocytosis and a CRP level of 500 mg/L, sepsis with a procalcitonin level of 27 ng/mL, and no impairment of renal or hepatic function (Table 1). Abdominal computed tomography (CT) revealed generalized fecal peritonitis due to perforation of a cecal tumor, which presented as a large heterogeneous mass with circumferential, irregular wall thickening measuring 25 mm in maximum thickness and extending over a height of 11.5 cm, with no suspicious pulmonary, hepatic, or bone lesions. These findings prompted an urgent surgical approach (Figure 1). 

**Table 1:** Laboratory tests showed the following results

Parameter	Value	Reference Range	Unit
White blood cell count (WBC)	17.6	4.0–10.0	×10³ cells/µL
Polymorphonuclear neutrophils (PNN)	9.7	1.5–7.0	×10³ cells/µL
C-reactive protein (CRP)	500	<5	milligrams per liter (mg/L)
Procalcitonin (PCT)	27	<0.5	nanograms per milliliter (ng/mL)
Serum creatinine (SCr)	0.9	0.6–1.2	milligrams per deciliter (mg/dL)
Alanine aminotransferase (ALT)	18	7–56	units per liter (U/L)
Aspartate aminotransferase (AST)	17	10–40	units per liter (U/L)

**Figure 1:**
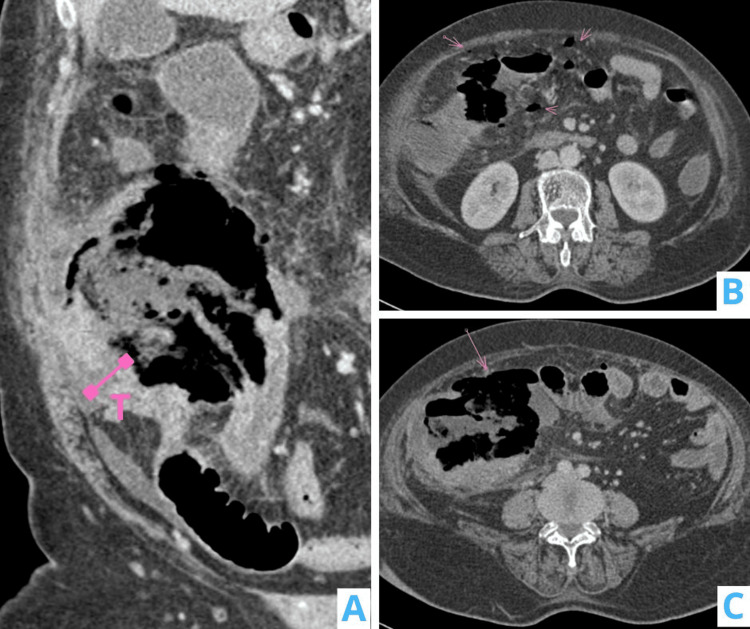
CT imaging of cecal perforation with pneumoperitoneum (A) Thickening "T" of the cecal wall with perforation, measuring 25 mm in maximum thickness. (B, C) Extraluminal gas "arrow" (pneumoperitoneum), consistent with perforation of the underlying tumor.

Intraoperatively, exploration revealed an extensive cecal mass involving the perforated ascending colon and associated with peritoneal implants. A right oncologic colectomy with double stoma was performed (Figure 2). The choice of this surgical strategy, with deferral of primary anastomosis, was guided by the patient's generalized purulent peritoneal collection and septic state, in accordance with damage-control surgery principles. The postoperative course was favorable after a two-day stay in the intensive care unit (ICU), and the patient was discharged on the eighth day after resumption of bowel function. 

**Figure 2:**
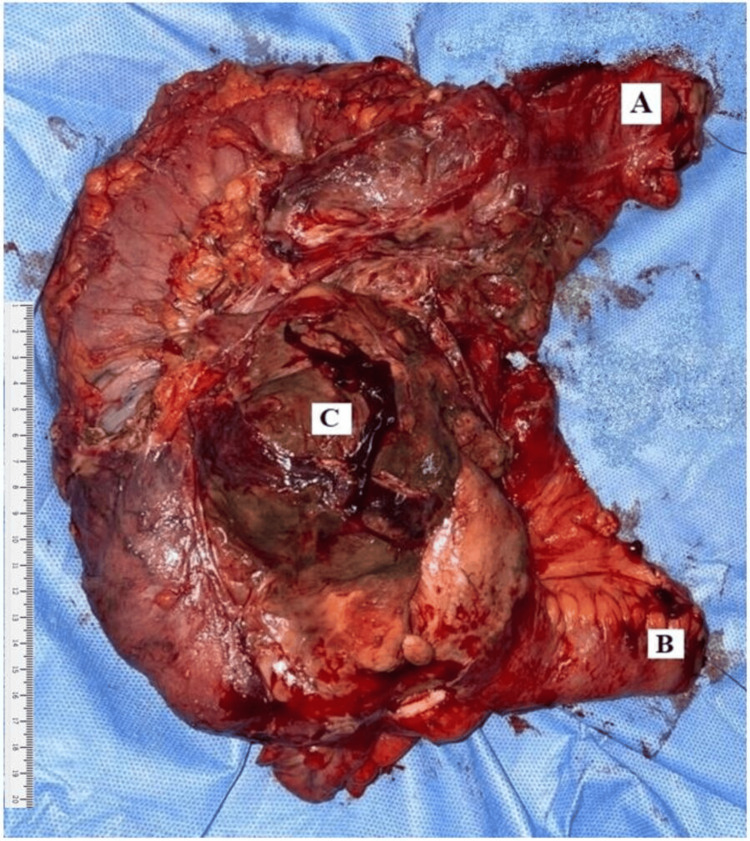
Macroscopic surgical specimen after right colectomy Macroscopic view of the surgical specimen following a right colectomy, with the tumor mass located in the cecal fundus. The specimen is labeled as follows: (A) cross-section of the transverse colon, (B) cross-section of the terminal ileum, and (C) the tumor mass itself.

Anatomopathological examination of the right colectomy specimen revealed diffuse large B-cell lymphoma, germinal center type (CD20+, CD79a+, CD10+, Bcl6 weakly positive, Bcl2 negative, CD3-, CD5-, cytokeratin negative), with a high proliferation index (Ki-67, 90%) and areas of tumor necrosis. The resection margins (ileal and colonic) were free of tumor (R0 resection). Thirteen lymph nodes were examined, all of which were free of tumor infiltration (13N-/13N). The appendix was not infiltrated by the lymphoma (Figure 3). The patient was referred to the hematology-oncology department for adjuvant management. Complete staging workup confirmed the absence of secondary localizations (stage IIE according to the Ann Arbor classification). The International Prognostic Index (IPI) score was 2 (age >60 years and stage IIE), classifying the patient in the intermediate-risk group. 

**Figure 3:**
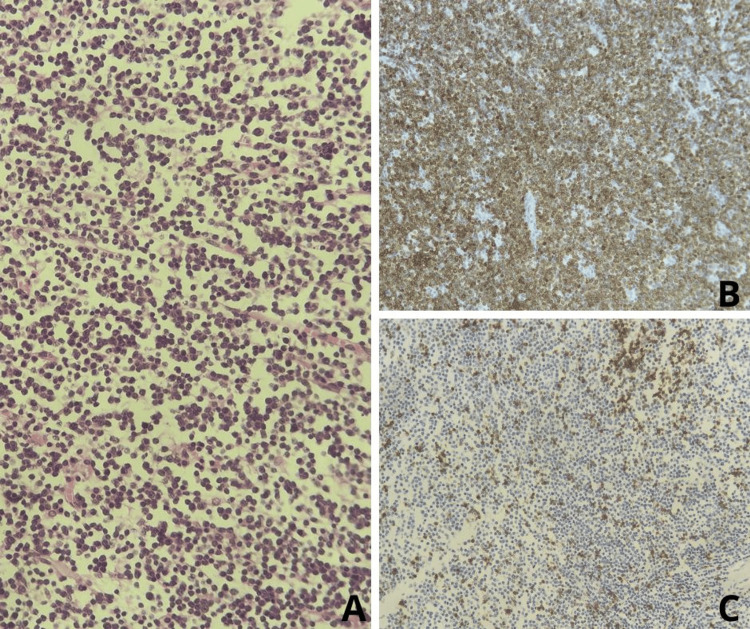
Histopathological and immunohistochemical features of diffuse large B-cell lymphoma (A) Diffuse lymphoid proliferation obliterating the normal architecture. The neoplastic cells exhibited vesicular nuclei, prominent nucleoli, and sparse, non-cohesive cytoplasm, supporting a diagnosis of lymphoid neoplasia (Hematoxylin-eosin staining, original magnification ×400). (B) Positive cytoplasmic staining for cluster of differentiation 79a (CD79a) in tumor cells (Immunohistochemical staining with DAB chromogen, original magnification ×200). (C) Negative staining for cluster of differentiation 3 (CD3) in tumor cells, confirming a B-cell phenotype (Immunohistochemical staining with DAB chromogen, original magnification ×200).

Treatment with immunochemotherapy based on rituximab, cyclophosphamide, doxorubicin, vincristine, and prednisone (R-CHOP) was initiated six weeks after surgery, following complete resolution of sepsis and satisfactory wound healing. The patient received four cycles of R-CHOP with good clinical and hematological tolerance. Interim imaging performed after four cycles of R-CHOP demonstrated a partial response according to the Lugano criteria, with a significant decrease in metabolic uptake at the colonic site but persistence of minimal residual activity. In the absence of disease progression and given the good clinical tolerance, the multidisciplinary team decided to continue treatment to complete six cycles, with reassessment planned at 12 months.

The patient's clinical course was as follows. Emergency surgery was performed on day 0, with ICU discharge on day 2 and hospital discharge on day 8 following resumption of bowel function. R-CHOP immunochemotherapy was initiated at week 6, and a partial response was documented on PET-CT evaluation at six months. The patient was informed about the rarity of her condition and the need for both emergency surgery and long-term oncological follow-up. She expressed satisfaction with the multidisciplinary care provided and remains motivated to continue her treatment. She has been actively involved in all therapeutic decisions and understands the importance of completing the planned six cycles of R-CHOP and subsequent surveillance.

## Discussion

We report a rare case of diffuse large B-cell lymphoma presenting with fecal peritonitis secondary to colonic perforation. Colonic perforation as the initial manifestation of lymphoma is exceedingly rare. In a large series of 146 patients with primary gastrointestinal lymphomas, only 3.4% presented with perforation [3]. Among colonic tumors requiring emergency surgery for perforation, lymphomas account for less than 1% [4]. To date, fewer than 20 cases of perforated colonic lymphoma have been reported in the English-language literature, mostly as single case reports. Our case adds to this limited body of evidence and highlights the importance of considering lymphoma in the differential diagnosis of perforated colonic tumors.

Analysis of previously reported cases reveals several important findings. Konishi et al. reported an 88-year-old man with transverse colon perforation treated by partial colectomy and colostomy, with a favorable short-term outcome [9]. Butterworth et al. published the largest systematic review, including 95 patients with abdominal catastrophes secondary to lymphoma, demonstrating that perforation is the most common presentation (69%), predominantly affecting the small bowel [10]. Diffuse large B-cell lymphoma (29%) and Burkitt lymphoma (28%) were the most frequent subtypes. Notably, patients with B-cell lymphoma were predominantly older, had new diagnoses, and presented with a balanced proportion of obstruction and perforation, consistent with our 70-year-old patient [10]. Ara et al. reported eight cases of intestinal perforation due to non-Hodgkin lymphoma, including only two colonic localizations (cecum and sigmoid colon), with a high mortality rate (37.5%) [11]. An exceptional case of colo-splenic fistula secondary to perforated colonic lymphoma was reported by Al-Zahir et al. in a 35-year-old man who remained disease-free at five years following en bloc resection and chemotherapy [12]. Our case adds to this limited literature by documenting a cecal diffuse large B-cell lymphoma with an exceptionally high proliferation index (antigen Ki-67, 90%) presenting with fecal peritonitis. Compared with previously reported cases, our patient's presentation with septic shock requiring intensive care reflects the severity of this complication, yet her favorable early postoperative course demonstrates that timely surgical intervention can be life-saving.

Several mechanisms may explain perforation in colonic lymphomas. First, transmural infiltration by lymphomatous cells weakens the colonic wall, making it susceptible to spontaneous or distension-induced rupture [6]. Second, rapid tumor growth can lead to central necrosis and subsequent perforation, particularly in aggressive subtypes such as diffuse large B-cell lymphoma [1]. Third, the tumor may cause partial obstruction with proximal colonic distension, increasing intraluminal pressure and precipitating perforation at or proximal to the tumor site [5]. In our patient, the stenosing cecal tumor with a high proliferation index (antigen Ki-67, 90%) likely combined these mechanisms, resulting in perforation and fecal peritonitis.

Emergency surgery for perforated colonic lymphoma presents specific challenges. The primary objectives are source control of sepsis and resection of the tumor for both treatment and diagnosis [8]. In our case, right oncologic colectomy with double stoma was performed due to the generalized purulent peritoneal collection and septic condition. This approach aligns with current recommendations that favor resection with temporary stoma over primary anastomosis in contaminated settings to reduce anastomotic leak risk [7]. The extent of surgical resection in lymphomas differs from that in adenocarcinomas. Since lymphomas spread via submucosal and lymphatic routes rather than direct extension, wide mesocolic excision with extended lymphadenectomy is not required [2]. However, complete macroscopic resection (R0) should be achieved when feasible, as it may improve outcomes [8].

Accurate histopathological diagnosis is crucial as it determines adjuvant treatment. In our case, immunohistochemistry confirmed diffuse large B-cell lymphoma of germinal center type (CD79a+, CD3-, Ki-67, 90%). The high proliferation index (>80%) is associated with more aggressive behavior and poorer prognosis, reinforcing the need for prompt adjuvant therapy [1]. The germinal center subtype, identified by immunohistochemical algorithms, generally carries a better prognosis than the activated B-cell subtype when treated with R-CHOP [10].

For localized diffuse large B-cell lymphoma (stage I-IIE), the standard treatment is R-CHOP immunochemotherapy with or without involved-field radiotherapy [10]. Complete resection (as achieved in our case) does not obviate the need for adjuvant chemotherapy, which reduces relapse risk from 50-60% to 20-30% [8]. In perforated cases, timing of chemotherapy is critical: it should be delayed until the patient has fully recovered from sepsis and surgery, typically four to six weeks postoperatively, to avoid immunosuppression during the vulnerable period [7]. Prognostic factors in colonic lymphoma include stage, International Prognostic Index (IPI), histologic subtype, proliferation index, and completeness of resection [3]. Our patient's age (70 years), high Ki-67 (90%), and perforated presentation place her in an intermediate-risk category, underscoring the importance of completing planned immunochemotherapy and close follow-up.

This report has limitations inherent to single case descriptions. Long-term follow-up beyond six months is not yet available to assess complete response and survival outcomes. Additionally, the optimal timing of chemotherapy after perforated lymphoma surgery remains debated and requires further study. Based on our experience and literature review, emergency laparotomy with oncologic resection and temporary stoma appears to be the preferred approach in contaminated settings. Extensive lymphadenectomy is unnecessary; the focus should be on complete tumor resection with negative margins. If lymphoma is suspected intraoperatively, tissue should be sent fresh to pathology for flow cytometry and cytogenetics. Multidisciplinary discussion involving surgeons, hematologists, and pathologists is essential postoperatively. Adjuvant R-CHOP should be initiated after complete recovery from sepsis and surgery, typically four to six weeks.

## Conclusions

This case underscores the need to consider colonic lymphoma in the differential diagnosis of perforated cecal tumors, particularly in elderly patients with nonspecific abdominal symptoms. Emergency colectomy serves both diagnostic and therapeutic purposes, allowing source control of sepsis and providing tissue for definitive histological diagnosis. Multidisciplinary management, involving surgeons, pathologists, and oncologists, is essential for optimizing outcomes. Timely adjuvant immunochemotherapy, typically initiated after recovery from surgery and sepsis, plays a key role in reducing relapse risk. Long-term follow-up remains necessary to assess complete response and relapse-free survival. Raising clinician awareness of this rare entity could facilitate earlier recognition and improve patient prognosis.
